# A digital hardware design for real-time simulation of large neural-system models in physical settings

**DOI:** 10.1186/1471-2202-15-S1-P21

**Published:** 2014-07-21

**Authors:** Murphy Berzish, Bryan Tripp

**Affiliations:** 1 Centre for Theoretical Neuroscience, University of Waterloo, Waterloo, Ontario, Canada, N2L 3G1

## 

The organization of neural systems reflects the specific complexities of the physical environments in which they operate. In order to address this relationship more directly, there is increasing interest in testing real-time neural simulations that interface with the physical world. We describe a new simulation approach that allows us to run large, sophisticated neural models on low-power embedded commodity hardware such as field-programmable gate arrays (FPGAs). A custom digital circuit was designed to approximate the collective outputs of populations of neurons that have correlated activity. These populations are taken to represent physical quantities in their spike rates. Information processing (e.g. function approximation) is taken to be determined by synaptic weights. This design is based on the Neural Engineering Framework (NEF), which bridges the gap between neural activity and higher-level behaviour [1,2].

Populations are grouped together on hardware execution components, which we call “population units”, that perform time-multiplexing in order to simulate 1024 populations per timestep. The population unit represents each population as a weighted sum of principal components of the neural tuning curves summed with a model of the associated high-frequency spike-related fluctuations. These principal components span the functions that weighted sums of spikes can approximate without being dominated by spike-related noise. Populations running on the same population unit use the same principal components, which saves memory and improves the speed of the simulation. Clustering is performed prior to simulation, which groups together populations which can be accurately represented by shared principal components.

The hardware does not need to be customized or regenerated in order to simulate different networks. It can be programmed with a network description generated by a compiler that operates as a backend to the Nengo simulator. (Nengo was used to run the Spaun model [2].) The design was implemented on an FPGA and was able to run simulations of up to 45 thousand populations of neurons (a realistic surrogate model of about 1-5 million point neurons) in real-time at 12-bit accuracy on a 1 millisecond timestep. Input and output to the hardware is over Gigabit Ethernet and can be collected from a PC running Nengo for simulation control and visualization. This implementation allows real-time approximate simulation of about the same scale as the largest real-time GPU simulations in Nengo, but using much less power. Furthermore, the FPGA chip is suitable for embedded applications such as mobile robots, cameras, etc. This work greatly facilitates simulation of an essential feature of neural systems, their embodiment and interaction with the physical world.
